# Effect of Waste Glass Addition as a Replacement for Fine Aggregate on Properties of Mortar

**DOI:** 10.3390/ma13143189

**Published:** 2020-07-17

**Authors:** Marcin Małek, Waldemar Łasica, Mateusz Jackowski, Marta Kadela

**Affiliations:** 1 Faculty of Civil Engineering and Geodesy, Military University of Technology in Warsaw, ul. Gen. Sylwestra Kaliskiego 2, 01-476 Warsaw, Poland; marcin.malek@wat.edu.pl (M.M.); waldemar.lasica@wat.edu.pl (W.Ł.); mateusz.jackowski@wat.edu.pl (M.J.); 2 Building Research Institute (ITB), ul. Filtrowa 1, 00-611 Warsaw, Poland

**Keywords:** by-product waste, glass cullet, recycling, eco-efficient mixture, slump flow, mechanical properties, compressive strength, flexural strength, split tensile strength

## Abstract

A responsible approach towards sustainable development requires the use of environmentally friendly, low-carbon, and energy-intensive materials. One positive way is to use glass waste as a replacement for fine natural aggregate. For this purpose, the effects of adding glass cullet to the mechanical properties of mortar were carried out. The glass aggregate made from recycled post-consumer waste glass (food, medicine, and cosmetics packaging, including mostly bottles), were used. This experimental work included four different contents of fine glass cullet (5, 10, 15, and 20 wt.% of fine aggregate). The compressive, flexural, and split tensile strengths were evaluated. Moreover, the modulus of elasticity and Poisson coefficient were determined. The addition of glass sand aggregate increases the mechanical properties of mortar. When comparing the strength, the obtained improvement in split tensile strength was the least affected. The obtained effect for the increased analysed properties of the glass sand aggregate content has been rarely reported. Moreover, it was determined that by increasing the recycled glass sand aggregate content, the density of mortar decreased. In addition, the relationships between the properties for mortar containing glass sand aggregate were observed.

## 1. Introduction

Due to significant emphasis on increasing sustainability and environmental protection, there is also a need to take this into account in construction practices. The building and construction sector were responsible for 36% of the final energy consumption and 39% of energy and carbon dioxide (CO_2_) emissions associated with the process in 2018. Of this, 11% was due to the production of building materials and products such as steel, cement and glass [1]. Cement production is an energy-intensive process that accounts for 5% of the industrial energy consumption and 3% of the total energy consumption worldwide [2]. Moreover, the production of one tonne of cement generates about 0.9 tonnes of CO_2_ by decarbonisation, which is released into an environment [3,4].

Meanwhile, concrete is the most commonly used building material in the world (the world’s production in 2012 amounted to 7 million m^3^ per year, i.e., three times more than wood and seven times more than steel per year [5]). According to industry statistics published in The Global Cement Report, 13th Edition [6], the consumption of cement in the world increased by 2.8% per cent to 4.08 million tonnes in 2019. Therefore, there is a need to look for materials as a replacement for cement or aggregate. Recycled materials are used to limited energy consumption by the concrete industry and the production of concrete as an environmentally friendly material [7,8,9,10]. The use of fly ash as a replacement for cement was studied by many researchers [11,12,13,14,15]. Gesoglu et al. [16] carried out the use of plastic powder as a replacement of cement. However, glass can be regarded as the most suitable as a sand and cement substation, due to its chemical composition and physical properties [17,18,19]. Glass powder as a cement replacement was investigated by many scientists [20,21,22]. Federico and Chidiac [23] studied the impact of glass cullet that was ground to a powder form for selected properties of cement mixes. Ankur and Randheer [24] investigated the mechanical parameters of concrete containing the addition of glass powder. Kou and Xing [25] analysed the same problem. They determined that fine milled glass powder had similar or slightly better mechanical parameters at later stages than fly ash and slag, but much less than silica fumes. At the same time, Mirzahosseini and Riding [20] used glass powder and investigated the effect of the glass colour and curing temperature on the pozzolanic reactivity of glass powder and properties of concrete. Moreover, the glass cullet used as aggregate in concrete was reported by [26,27,28]. Mohammadinia et al. [29] used glass and polymer aggregates as a replacement for gravel aggregate for the production of concrete class C50/60. Limbachiya [30] and Taha [31] determined that the use of recycled glass as a replacement for sand reduced the workability of concrete due to a lack of its fine proportion. The influence of different sizes of glass aggregates on the properties of self-compacting mortar was carried out by Ling and Poon [31]. Meanwhile, Yousefi et al. [32] investigated the impact of different particle sizes of expanded glass aggregate (EGA) on the compressive strength. Bostaci et al. [33] used 15% of glass sand from recycling with a particle size of up to 5 mm and obtained an 8.1% decrease in compressive strength. Ling and Poon [31] determined a 9.6% decrease in the compressive strength of concrete with 60 wt.% addition of glass aggregate size of 0–2.36 mm and 40 wt.% addition of glass aggregate size of 2.36–5 mm. According to Limbachiya et al. [34], using recycled glass sand in concrete mixes up to 15% indicates comparable mechanical properties. Kou and Poon [35] achieved a negative effect on bonding and a reduction in strength by using glass as a coarse aggregate in concrete. The same phenomenon was observed by Tittarelli et al. [36]. According to Lee et al. [37], the use of glass aggregates with a particle size of 2.36–5 mm or 5–10 mm in concrete causes alkali-silica reaction (ASR) expansion and the strength reduction. This limits the use of recycled glass in concrete [38,39]. The most previously reported results showed an increase in ASR expansion with more glass sand content [40,41,42]. However, Du and Tan [39] determined that by increasing the addition of glass sand, ASR expansion decreased for green and brown glass sand mortars and mortar containing clear glass aggregate up to 25%. The same trend was observed by Ismail and Al-Hashmi [43] and Saccani and Bignozzi [44], who observed a decrease in ASR expansion for mortar with glass sand content up to 20% and 35%, respectively. In addition, ASR expansion increased by increasing the glass sand size at each test stage [39]. The vast spread of reported results may be related to the micro-cracks in glass particles [45]. Tan and Du [39,46] reported that the most significant decrease in compressive strength was observed for concrete with 25%, 50%, 75%, and 100% of glass aggregate made from clear glass, while the smallest was for green glass (except concrete with 25% addition of green glass particles, for which an increase was obtained). At the same time, more micro-cracks in the clear glass particles, very few micro-cracks in the green glass particles, and almost no micro-cracks existing in the brown glass particles were observed [39,46]. A number of formed micro-cracks can be dependent on the crushing process [45,47] and perhaps the post-cracking strength of glass [48,49,50].

Castro and Brito [51] reported that the addition of 5–20 vol.% of recycled glass sand to concrete improved the long-term carbonation resistance due to the improvement of its pore structure. Yu et al. [52] also determined that the use of glass and steel slag aggregate improved the mechanical properties of concrete. Other scientists [20,53] obtained an increase in the long-term compressive strength, but for concrete/mortar with fine ground glass powder. Based on the above, the influence of the addition of a glass sand aggregate for improved mechanical properties of concrete/mortar has rarely been reported. This effect was achieved for concrete/mortar with low glass cullet content up to 20–35% (most often 20 or 25%), but almost every time it was the lowest content tested. Thus, this study aimed to determine the effect of low glass sand content up to 20% of glass on the material and mechanical properties of mortar. Unlike other scientists, the addition of 5%, 10%, 15%, and 20% glass sand aggregate made from post-consumer waste glass as a replacement for fine aggregate was used. In addition, the relationships between material and mechanical properties of mortar with glass sand aggregate were demonstrated.

The test results make a significant contribution to the possibility of using post-consumer glass in a mortar and/or concrete, thereby reducing landfills.

## 2. Materials

### 2.1. Specimen Preparation

The materials used in this study were Portland cement, tap water and a polycarboxylate superplasticiser.

#### 2.1.1. Cement

The industrial Portland cement (Górażdże, Opole, Poland) was CEM I 52.5R, according to PN-EN 197-1:2011. Its chemical composition and physical properties were determined according to PN EN 196-6:2011, are shown in Table 1. The compressive strength of cement was investigated as per PN EN 196-1:2016-07 (Table 2).

#### 2.1.2. Aggregate

**Figure 1:**
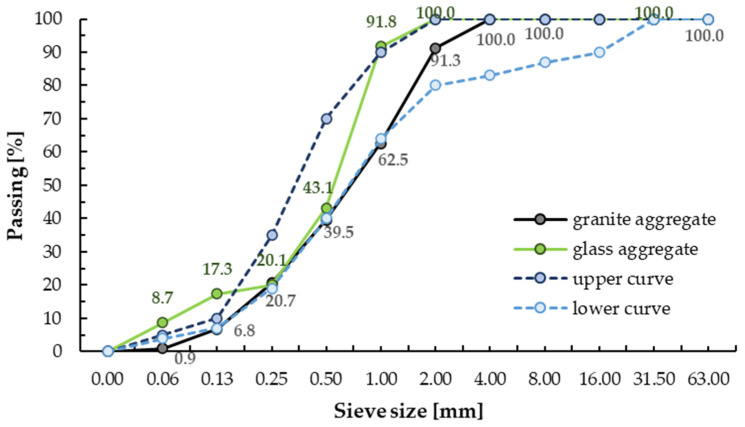
Gradation curve of crushed aggregate.

The crushed granite aggregate (GA) with fractions of 0–4 mm was used. Granite aggregate (grain size index *C_U_* = 7.1 and *C_C_* = 2.3) is heterogeneous and well compacted, see Figure 1. The background is the upper and lower curves determined in accordance with the PN-EN 12620+PN-B-06265 standard for natural aggregate with fraction up to 4.0 mm.

**Figure 2:**
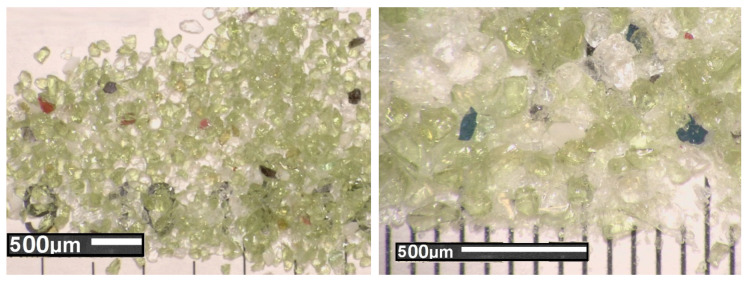
Light microscope (OPTA-TECH STX12, Opta-Tech, Warsaw, Poland) images of glass aggregate.

In addition, glass cullet with fractions of 0–2 mm was used to replace the granite aggregate. The glass sand aggregate (GSA) was made from post–consumer waste glass (food, medicine, and cosmetics packaging, including mostly bottles) as a result of mechanical laboratory crushing. The recycled aggregate was washed after crushing to remove any dust. The glass cullet was then sieved and sorted into two size classes according to its gradations (0.0–0.9 mm and 0.9–1.5 mm). Based on experience, the glass aggregate was selected so that the ratio of 0.9–1.5 mm fraction to 0.0–0.9 mm fraction was 1.55. The gradation curve of glass aggregate is shown in Figure 1. The crushed glass cullet exhibited sharp edges and rougher surface texture, see Figure 2. The specific density and Mohs hardness scale of glass aggregate was approximately 1.6 kg/m^3^ and 6–7, respectively. Table 1 presents the chemical and physical properties of glass cullet.

#### 2.1.3. Superplasticiser

A superplasticiser based on modified polycarboxylate ethers (melamine and silanes/siloxanes) was used to obtain the required workability. The polymer admixture was added to reduce the amount of water.

### 2.2. Mix Composition

Five different types of mortar mixtures (four mixtures with and one without glass aggregate) were produced. The fine aggregate was crushed granite aggregate. The glass sand content was approximately 5%, 10%, 15%, and 20% by mass of the total aggregate (granite + glass cullet), see Table 3. For reference, a base mix without glass cullet was made. A constant *w_eff_/c* = 0.49 ratio was used for all mixes. The superplasticiser was used at a constant ratio of 1.0% of the mass of the cement in all mixes. The admixture content was selected experimentally.

### 2.3. Mix Production

Glass cullet of two fractions (0.0–0.9 mm and 0.9–1.5 mm) was mixed for three minutes. Next, the crushed granite aggregate (dried to constant weight) was added and all components were mixed for ten minutes. To this, cement was added. After six minutes of mixing the components, tap water mixed with the superplasticiser was added. All components of the mortar were mixed for ten minutes. The entire mixing time lasted a maximum of 22 min. Samples were produced in laboratory conditions (21 °C and 50% humidity). After pouring the mixture into plastic moulds (specimens with dimensions of 150 × 150 × 150 mm, 100 × 100 × 500 mm, 40 × 40 × 160 mm, and Φ150 × 300 mm), the mix was compacted using a vibrating table. Twenty-four hours after pouring the mix into the mould, the upper layer of the sample was protected against evaporation of water by covering the moulds with an impermeable foil. Samples were demoulded after 36 h. Samples up to 28 days were stored in a climate chamber at a temperature 20 ± 2 °C and humidity 90%.

## 3. Methodology

### 3.1. Test on Mix

**Figure 3:**
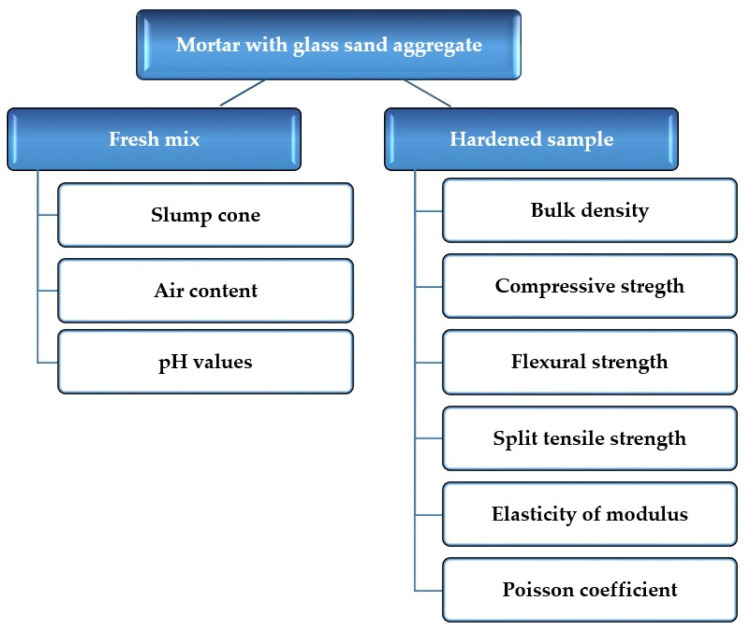
Schema diagram of properties determined in tests.

The slump cone and air content of the fabricated fresh mix and pH value of liquid phase extracted from fresh mixtures were investigated immediately after mixing process of ingredients. Also, the pH value of the liquid phase extracted from fresh mixtures was tested. A schema diagram of tests performed is shown in Figure 3. Five specimens were tested for each mortar mix.

#### 3.1.1. Slump Cone

The workability of a fresh mixture of mortar was measured using a slump cone (Merazet, Poznań, Poland) according to PN-EN 12350-2.

#### 3.1.2. Air Content

**Figure 4:**
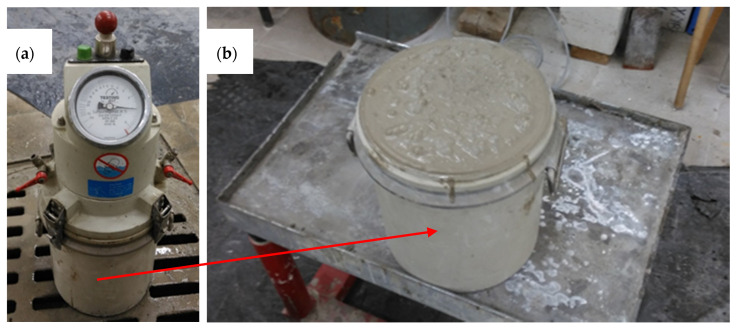
The pressure method (**a**) pressure porosimeter (Merazet, Poznań, Poland), (**b**) bottom porosimeter container filled with a mixture of mortar with 5% of glass aggregate.

The air content of mortar mixes was investigated per PN-EN 12390-3:2019-07. The pressure method was used, see Figure 4. The sample was placed in an 8 L vessel. Then, the vessel was filled with water under pressure. After equalising the pressure, the manometer value was read.

#### 3.1.3. pH Value

The pH value was determined for the liquid phase extracted from a fresh mix at 10 mL, according to PN-EN 1015-3. The pH meter (Testo, Pruszków, Poland) was equipped with a suspension electrode. The pH measurement was taken at three minutes. After this time, the value was noted.

### 3.2. Test on Hardened Mortar

The tests on hardened mortar were carried out after 28 days of the curing process. A schema diagram of tests performed is shown in Figure 3. Five specimens were measured for each mortar mix to determine material properties and ten specimens to determine mechanical properties.

#### 3.2.1. Material Properties

The bulk density of hardened samples of mortar was determined using 150 × 150 × 150 mm standard cubes according to PN-EN 12350-7:2011.

#### 3.2.2. Mechanical Properties

The mechanical properties of the mortar were measured after 28 days of curing. The schema diagram of tests performed, as shown in Figure 3. The samples were tested using a Zwick machine (Zwick, Ulm, Germany) (force range 0–5000 kN).

##### Compressive Strength

Compressive strength was determined using standard samples of 150 × 150 × 150 mm, according to PN-EN 12390-3:2019+AC:2012.

##### Flexural Strength

**Figure 5:**
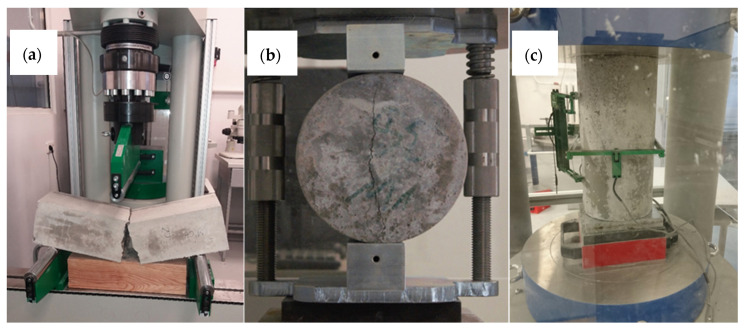
Photographic documentation of tests carried out: (**a**) flexural strength test, (**b**) split tensile strength test, (**c**) elastic modulus test.

Flexural strength was tested in a three-point bending set-up (Zwick, Ulm, Germany) with beams 100 × 100 × 500 mm and 40 × 40 × 160 mm, according to PN-EN 12390-5:2011. The nominal distance between the supports was 300 mm. The rollers allowed for free horizontal movement, see Figure 5a.

##### Split Tensile Strength

Split tensile strength was tested on cylinder-shaped samples with 150 mm diameter and 300 mm height, according to PN-EN 12390-6:2011. The example sample is presented in Figure 5b. The sample was placed on steel supports laterally and the “Brazilian Test” was done.

##### Modulus of Elasticity and Poisson Coefficient

The modulus of elasticity and Poisson ratio were determined according to PN-EN 12390-13:2014-02 with cylindrical specimens of 150 mm in diameter and 300 mm in height. Two electrical resistance strain gauges with 100 mm measurement length were bonded on two opposite sides of the specimens at mid-height. The stress-strain characteristic was recorded for the evaluation of the elastic modulus. In order to ensure parallelism, surfaces directly exposed to compressive stress were ground. Longitudinal and transverse linear displacements were measured using Epsilon extensometers (Epsilon, Jacson, USA). A frame model (recording transverse linear displacement values) and an axial jaw model (recording longitudinal linear displacement values) were used. The samples were subjected to three loading and unloading cycles in the lower and upper-stress range depending on the characteristic compressive strength of the composite. Referring to the recorded values of linear displacements and the lengths of the measuring bases of the devices, deformation values for the lower and upper stress range in mortar samples were determined.

## 4. Results

### 4.1. Fresh Properties

**Figure 6:**
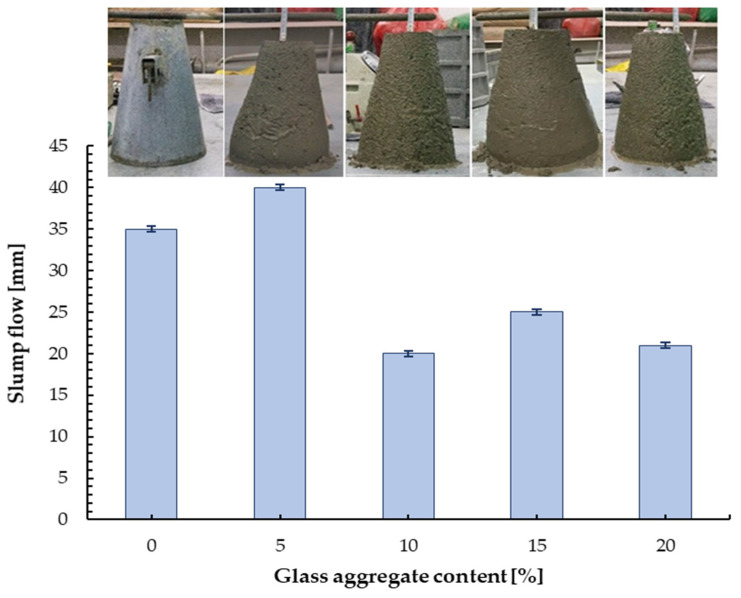
Slump cone test results after 150 s.

For all mortar mixtures, no segregation or bleeding was observed during mixing and casting. Fresh properties of the mortar mixes were established through slump test and pH values. The results of the slump cone test are shown in Table 4. The presented values are average values of the five samples for each mix. It was determined that all mixes were within slump cone class S1. The addition of glass cullet to mortar reduced its workability, except for a mix with a 5% glass cullet, see Figure 6. With the addition of 10 and 20 wt.% of glass cullet, the decrease in slump cone was about 40% compared to the base mix (without glass content).

The results of the pressure pore test of mortar mixes are shown in Table 5. The air content of the mortar mix with fine glass aggregate was between 1.8% and 2.4%. This is consistent with the observations carried out by other scientists. Tan and Du [46] obtained an air content between 3.0 and 3.5 for concrete with 25, 50 and 75% fine glass aggregate, regardless of the type of the glass. Park et al. [55] determined that an air content in concrete increased from 12.2% to 41.4% for concrete with 30%, 50%, and 70% of glass sand compared to normal mortar and almost 200% for concrete with 100% of clear glass sand. They used glass aggregate with a sharper edge (see Section 2.1.2) and high aspect ratio, which enables more air to be retained on the surface of a glass particle. Also, the slightly higher air content of concrete containing 20% of GSA was observed, while the obtained differences of air content for mortar with 0% to 15% of glass cullet were practically within the measuring error limit. According to Ling and Poon [31], a large amount of glass aggregate leads to an increase in bleeding and a more porous microstructure, because glass, by its nature, is an impermeable material, resulting in lower air voids contained in the mortars. Moreover, Ling and Poon determined that the smooth surface of glass aggregate causes lower air content in the mortar. While the irregular shape of GSA as in this study results in a larger relative surface area that trap more air in the mortar matrix. Also, Topçu and Canbaz [27] attributed the change in air content to the geometry of crushed waste glass.

It can be observed that the pH value of the liquid phase extracted from the fresh mixtures slightly increased with the addition of glass sand to the mix from 3.5% to 4.0% compared to the base mix (Table 4). The impact of glass aggregate content in a range of 5% to 20% of GSA on pH value is irrelevant. The obtained differences were within the measuring error limit.

### 4.2. Hardened Properties

Table 5 presents the results obtained for its material and mechanical properties. The presented values are average values of ten samples for each mix. From the obtained experimental data, the relationship between material and mechanical properties had been drawn along with a few developed empirical equations for finding the respective property of hardened mortar with the addition of glass sand as a replacement for fine traditional aggregate.

#### 4.2.1. Dry Density

**Figure 7:**
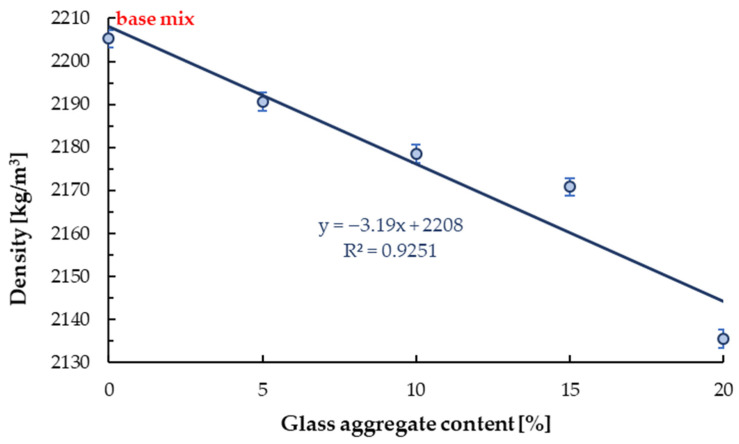
The impact of glass cullet ratio on the density of mortar with GSA.

The dry density results are given in Table 5. With the increasing of glass sand content, the density of mortar decreased linearly, but the effect of glass sand addition is slight, see Figure 7. The reduction in hardened density is due to the lower specific gravity of the glass cullet than the granite sand. With the addition of 5%, 10%, 15%, and 20% glass cullet, mortar density decreased by 0.6%, 1.2%, 1.5%, and 3.2% compared to the reference sample. The same effect of glass addition on density was observed by other scientists [55,56]. Park et al. [55] indicated a linear relationship between GSA content and density. Meanwhile, Lee et al. [56] determined that for concrete with 25% of glass sand with a particle size lower than 1.18 mm decrease in density was 0.8%. Meanwhile, for 25% of glass sand with a particle size lower than 2.36 mm, a 0.4%decrease in density was determined. Based on the above and on the results obtained in this study, it can be concluded that the addition of glass sand with a content of up to 20–25% slightly affects the density of mortar/concrete.

#### 4.2.2. Compressive Strength

**Figure 8:**
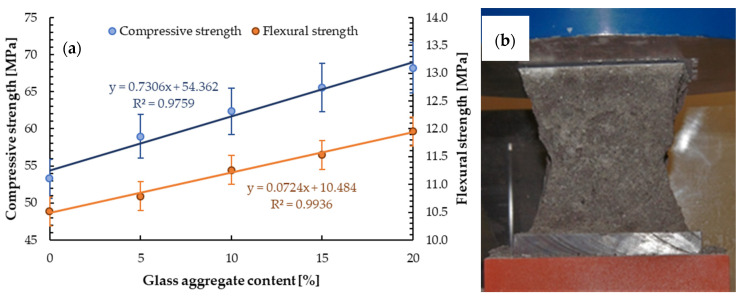
(**a**) Compressive and flexural strength depending on GSA ratio, (**b**) sample destruction.

Table 5 presents the test results of 28 days compressive strength for samples with the addition of glass aggregate. The compressive strength increased linearly with increasing glass sand content, see Figure 8a. With the addition of 5%, 10%, 15%, and 20 wt.% glass aggregate, the increase in strength compared to the reference sample (*f_c_* = 53 ± 1 MPa) was 11.3%, 17.0%, 24.5%, and 28.3% respectively.

The same trend was observed by Lee et al. [56] for concrete with fine glass aggregate with a particle size of 0–0.6 mm. They obtained 5.1%, 15.2%, 24.2%, and 34.3% increase in compressive strength compared to reference samples for concrete with 25%, 50%, 75%, and 100% of glass aggregate, respectively. Chung et al. [57] obtained about 19% higher compressive strength for concrete containing glass aggregates prepared from the brown soda-lime waste glass with a size of 0–4 mm compared to plain concrete. Tan and Du [46] determined a 1.6% increase in compressive strength for concrete with a 25% addition of green glass with a particle size of 0–4 mm, but 95% aggregate with a particle size of 0–2 mm was. Meanwhile, Bajad et al. [58] determined an increase in compressive strength by increasing the addition of glass powder as a cement replacement up to 20% before decreasing. The form of destruction of the sample is the same as for ordinary mixture (Figure 8b).

#### 4.2.3. Flexural Strength

The results of the flexural test samples with glass sand aggregate as replacement for sand are shown in Table 5. Figure 8a presents the linear relationships between flexural strength and glass sand content. It can be observed that with the addition of 5%, 10%, 15%, and 20 wt.% of glass sand aggregate flexural strength increased compared to a reference sample (*f_tk_* = 10.5 ± 0.3 MPa) by 2.9%, 7.6%, 9.5%, and 14.3%, respectively. Also, Tan and Du [46] determined a slight increase in flexural strength of concrete with a 25% addition of glass sand made from green glass (1.5%) compared to plain concrete. Chung et al. [57] obtained about an 18% higher flexural strength for concrete containing glass aggregates prepared from the brown soda-lime waste glass with a size of 0–4 mm compared to plain concrete. For other analysed mortars with other types of glass and different contents of glass sand, a reduction in flexural strength was obtained. Ling and Poon [31] reported about a 30% decrease in 28 days for the flexural strength of concrete with 60 wt.% addition of glass aggregate size of 0–2.36 mm and 40 wt.% addition of glass aggregate size of 2.36–5 mm.

#### 4.2.4. Split Tensile Strength

**Figure 9:**
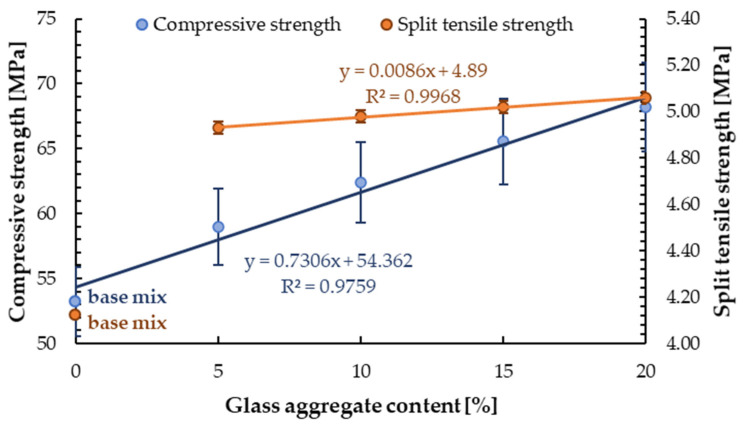
Compressive and split tensile strength of samples with GSA depending on GSA content.

The results of split tensile strength for samples with the addition of 5, 10, 15 and 20 wt.% of glass sand aggregate (GSA) are shown in Table 5. The linear relationship between split tensile strength and glass sand content was obtained, see Figure 9. With the addition of 5%, 10%, 15%, and 20 wt.% of GSA, the increase in split tensile strength compared to the reference sample (*f_r_* = 4.12 ± 0.05 MPa) was 19.7%, 20.9%, 21.8%, and 22.8%, respectively. It was observed that the difference in strength for the reference sample and the sample with the lowest addition of glass cullet was significant. However, although a further increase of glass sand content in mortar caused an increase in strength, this effect was not significant. For a 15% difference, the addition of a glass cullet (between 5% and 20% of GSA), increased the split tensile strength by about 3%. Thus, the obtained differences were practically within the measuring error limit. The increase in split tensile strength is consistent with the observations of Tan and Du [46]. They obtained a 5.4% increase in split tensile strength for mortar containing 25% glass sand made from green glass and a decrease for higher additions of GSA.

#### 4.2.5. Elastic Modulus and Poisson Coefficient

The obtained results for a study into the moduli of elasticity were 29 ± 1 to 33 ± 2 GPa (Table 5). Tan and Du [46] determined similar values. They reported that static moduli of mortar varied from 23 to 30 GPa for mortar with design strength of 50 MPa. The obtained elastic modulus of mortar with the addition of 5, 10, and 15 wt.% GSA were insignificantly lower compared to the reference sample (*E* = 32 ± 1 MPa). Tan and Du [46] determined about a 1% reduction in the modulus of elasticity for mortar containing 25% of glass sand made from the green glass. In this study, for an addition of 20 wt.% glass sand, the modulus of elasticity was about 3% higher than for the base mix. The same phenomenon was observed by Tittarelli et al. [36], but for coarse glass aggregate. This may be due to the fact that the modulus of elasticity for glass is higher than sand [59].

The results of the Poisson coefficient are presented in Table 5. Addition of glass aggregate in the range from 5 to 20 wt.% of fine aggregate did not affect the Poisson coefficient. For higher contents of glass aggregate, a greater spread of results was obtained.

## 5. Discussion

**Figure 10:**
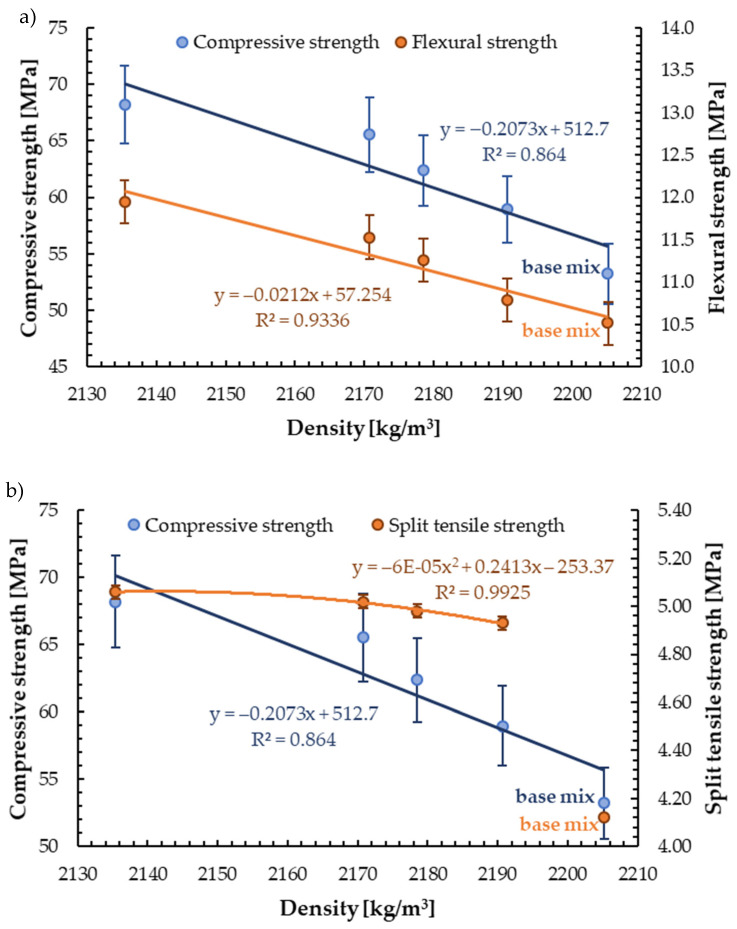
Effect of density on the mechanical properties of mortar containing glass sand aggregate, (**a**) relationship between density and compressive/flexural strength, (**b**) relationship between density and compressive/split tensile strength.

In this study, higher mechanical properties for mortar with 5%, 10%, 15%, and 20% of recycled glass sand aggregate than for reference mix (without GSA) were obtained, which is rarely reported in the literature. The increase in mechanical properties of mortar containing GSA with a decreasing trend in hardened density was determined. These correlations are linear, except for the correlation between split tensile strength and density, which is exponential (Figure 10). The rate of increase in flexural strength of mortar containing glass sand obtained in this study (11%) was significantly lower than that of compressive strength (17%). Tan and Du [46] obtained almost equal increase rates for both strengths. Compared with both strengths, the obtained increase in split tensile strength (3%) was the least affected. The coefficients of determination *R*^2^ for all relationships were achieved above about 0.9, which means a very good fit.

**Figure 11:**
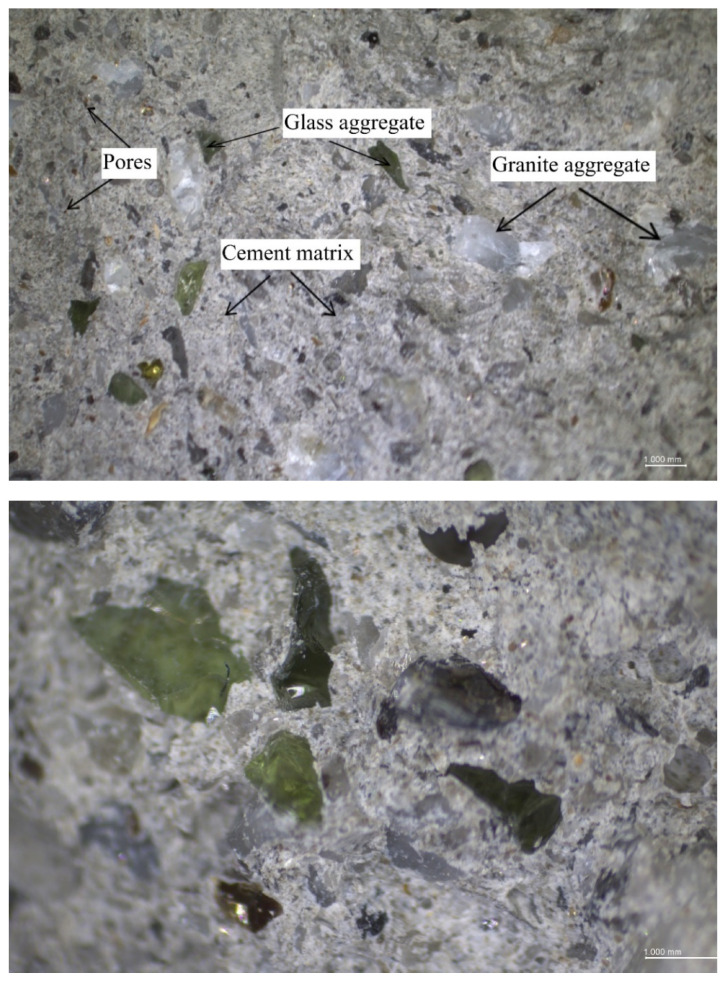
Light microscope images of microstructure of mortar with 10% of glass cullet.

**Figure 12:**
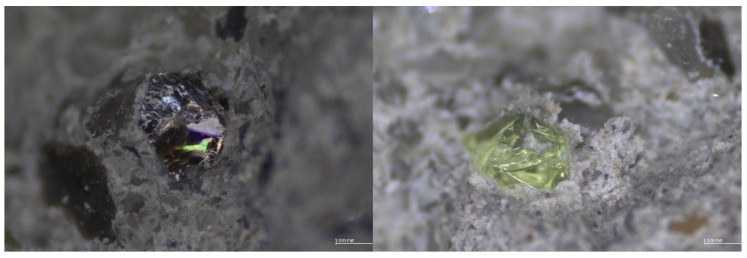
Light microscope images of individual glass particles of glass cullet in the cement matrix.

The observed improvement of compressive strength despite a decrease in dry density is an interesting phenomenon. This can be explained by the use of lighter and harder glass sand than the replaced sand aggregate. The density of granite ranges from 2.65 to 2.75 g/cm³, while the used glass cullet is about 60% lighter. However, the granite hardness on the Mohs scale is from 5.5. to 7 depending on the quartz and feldspar content. Mohs hardness of the used glass sand aggregate was 6–7 (see Section 2.1.2). The glass aggregate that was used has sharp edges and rougher surface texture, which resulted in good adhesion to the cement matrix, see Figure 11. The same conclusions regarding the influence of bond strength at the interfacial transition zone (ITZ) between the glass aggregate particles and the cement matrix on the compressive strength were reported by other scientists [35,46,60]. Mostly it is stated that the inclusion of a glass sand aggregate formed a weak adhesion between the interface of the glass sand and the cement paste matrix. However, this reduction was most often caused by the relatively smoother surface of GSA particles used and a series of its stepped fractures [31] that initiate expansive ASR gel [39]. Figure 12 presents selected individual glass particles. The used glass cullet, for the most part, was made of green glass, which is characterised by very few micro-cracks in the glass particle. The micro-cracking was not seen in particles using an LM microscope (OPTA-TECH STX12, Opta-Tech, Warsaw, Poland) at 28 days. This is consistent with the observations of Du and Tan [39], who only detected cracks on day 63. As in their research, no ASR gel was observed in current studies.

Moreover, this effect of an increase in mechanical strength could be attributed to the pozzolanic reaction of fine particles of the used glass aggregate [45,56,61]. Other scientists [23,62,63] have come to the same conclusion. Shi et al. [64] determined that the strength activity indices of fine glass powder with particle sizes ranging from 40 to 700 μm were 74% at 28 days, but 70% at seven days. Yamada et al. [65] determined the critical particle size for the occurring pozzolanic reaction at 0.15–0.30 mm, while Jin et al. [38], Idir et al. [66] and Xie et al. [67] at 0.6–1.18 mm. This glass particle size is similar to the glass particle size used in this study (0–1.5 mm). Glass aggregate with a 0–0.5 mm fraction was about 43% and 0–1.0 mm about 92% (Figure 1). Due to an occurring pozzolanic reaction the microstructure is more densified, thereby reducing the porosity and enhancing the bond strength at the interfacial transition zone (ITZ) between recycled aggregates particles and cement paste matrix [39,68], see Figure 11 and Figure 12.

**Figure 13:**
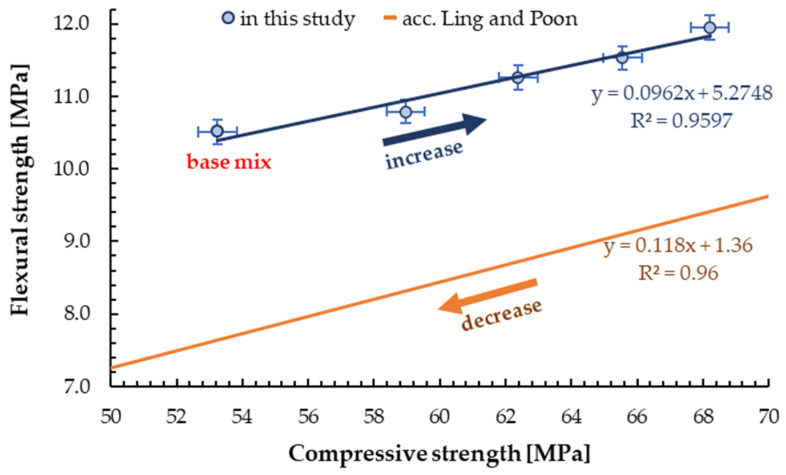
The correlation between compressive and flexural strength for samples with GSA.

**Figure 14:**
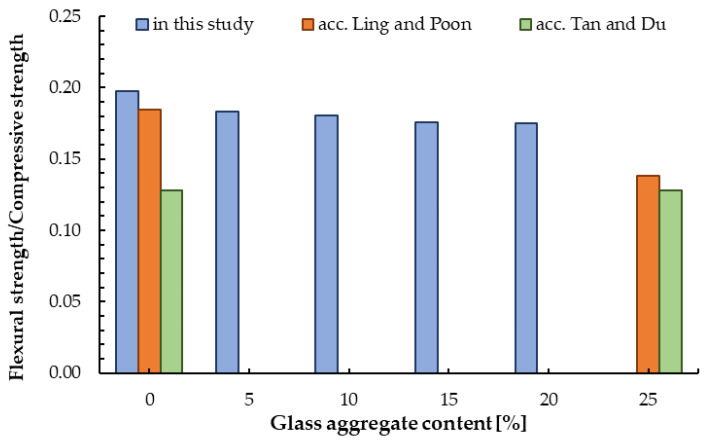
The ratio of tensile strength to compressive strength for samples with GSA.

When analysing new materials, it is imperative to determine the correlations between the appropriate mechanical properties with particular emphasis on compressive strength as the most important by design mechanical parameter. The relationship between compressive and flexural strength was presented in Figure 13. The background is a curve formed according to a pattern developed by Lind and Poon [31]. A very similar trend can be observed, but the strengths demonstrated in this study was higher than the reference samples. While Lind and Poon obtained strengths lower compared to the base mix. Moreover, they determined that the flexural strength was found to be about one-fourth to one-sixth of the compressive strength. Other scientists present the ratio of tensile strength to compressive strength for mortar/concrete containing glass aggregate ranging from about 13% to 25% [46,55,58]. The results achieved in this study, in a range from 18% to 20%, are part of this trend. The results for mortar/concrete with the addition of glass aggregate made from green glass with a compressive strength of 50 MPa are shown in Figure 14.

**Figure 15:**
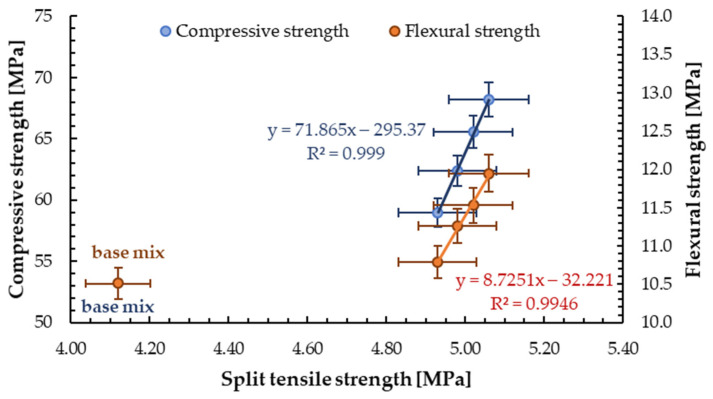
Relationships between mechanical properties of mortar containing glass sand aggregate.

The correlations between other mechanical properties such as compressive, flexural, or split tensile strength of mortar with GSA addition are shown in Figure 15. Linear relationships between split tensile and compressive/flexural strength can be observed. For analysed correlations, the obtained mechanical properties for mortar containing glass sand aggregate and reference sample (without GSA) were different. The coefficients of determination *R*^2^ for all relationships were achieved above 0.98, which means an extremely good fit.

## 6. Conclusions

The aim of the study was to assess the possibility of using a glass sand aggregate made from post-consumer waste glass (food, medicine, and cosmetics packaging, including mostly bottles) in concrete that is difficult to recycle and is stored in large quantities in landfills. The three different contents of glass cullet with a sharp edge and rougher surface texture were carried out as a replacement for fine aggregate in mortar. It was 5, 10, 15 and 20 wt.% of glass sand aggregate (mostly of the green glass cullet). Based on the results of this experimental investigation, the following key conclusions can be drawn:

- A decrease in the slump cone with the addition of recycled glass aggregate was observed, except 5 wt.% of GSA. However, all mixes were within slump cone class S1.
- The air content of mortar with fine glass aggregate was about 2%. The slightly higher air content of concrete containing 20% of glass content was observed, while the obtained differences of air content for mortar with 0% to 15% of glass cullet were practically within the measuring error limit.
- With the increasing of glass sand content, the density of mortar decreased, but this effect is inconsiderable (0.7–3.2% compared to the reference sample). The reduction in hardened density is due to the lower specific gravity of the glass cullet than the granite sand.
- With the addition of 5, 10, 15 to 20 wt.% glass aggregate, the increase in compressive, flexural, and split tensile strength of mortar with GSA compared to the reference mix were ranging about from 11% to 29%, 3% to 14% and 20% to 23%, respectively. The least increase in strength of mortar containing recycled glass aggregate was obtained for flexural strength, while the highest for compressive strength. The increase in mechanical properties was probably achieved thanks to the use of fine glass aggregate particles (0–1.5 mm), which enhance the aggregate-cement matrix bonding strength and the use of green glass aggregate with higher Mohs hardness. Each time, the greatest increase in strength was obtained for green glass with a small particle size up to 20%.
- The obtained elastic modulus of mortar with the addition of 5, 10 and 15 wt.% GSA were insignificantly lower compared to reference samples. While for the addition of 20 wt.% glass sand, the modulus of elasticity was about 3% higher than for the base mix. This may be due to the fact that the modulus of elasticity of glass is higher than sand.
- Addition of glass aggregate in the range from 5 to 20 wt.% of fine aggregate did not affect Poisson ratio. With a higher content of glass aggregate, a greater spread of results was obtained.
- For all analysed properties, relationships between it and the glass aggregate content were linear. Also, analysed mechanical properties were directly proportionate to the density of mortar with fine glass aggregate, expect flexural strength. The coefficients of determination *R*^2^ for all relationships were achieved above 0.8, which means a good fit.
- The relationships obtained in this study were rarely found in the literature. Therefore, they should be verified in further tests, taking into account other glass sand aggregate contents, its particle sizes, and in relation to the resulting alkali–silica reaction.

Based on the above, it seems that the increase in mechanical properties of mortar/concrete with the addition of glass sand is probably resulting from the pozzolanic activity. This will be the subject of further research, in which tests for other types of cement, other types of glass waste and its mixes, and different contents and the size of the aggregate particle, with particular emphasis on long-term fatigue tests are planned to be carried out.

## Figures and Tables

**Table 1:** Chemical composition of cement and glass cullet [54].

Compositions		SiO_2_	Al_2_O_3_	Fe_2_O_3_	CaO	MgO	SO_3_	Na_2_O	K_2_O	TiO_2_	Cl
Unit (vol.%)	cement	19.5	4.9	2.9	63.3	1.3	2.8	0.1	0.9	-	0.05
	glass	70.0–74.0	0.5–2.0	0.0–0.1	7.0–11.0	3.0–5.0	-	13.0–15.0 *	-	0.0–0.1	-

* Na_2_O equivalent.

**Table 2:** Physical properties of cement.

Specific Surface Area [m^2^/kg]	Specific Gravity [kg/m^3^]	Compressive Strength after Days [MPa]		
		2 days	7 days	28 days
**400**	3090–3190	40–48	53–65	66–76

**Table 3:** Mix proportions (1 m^3^).

Mix Symbol	Cement [kg]	Water [kg]	Admixture [kg]	Granite Aggregate [kg]	Glass Cullet [wt.% of Total Aggregate]	Glass Cullet [kg]
Base mix	511	250	0.51	1535	0	0
M1				1458	5	77
M2				1382	10	154
M3				1305	15	230
M4				1228	20	307

**Table 4:** Fresh properties.

Mix Symbol	Glass Cullet [wt.% of Total Aggregate]	Slump Cone [mm]	Air Content [%]	pH Value [-]
Base mix	0	35 ± 3	2.1 ± 0.2	12.03 ± 0.03
M1	5	40 ± 4	1.8 ± 0.2	12.50 ± 0.03
M2	10	20 ± 3	2.0 ± 0.1	12.45 ± 0.05
M3	15	25 ± 4	2.1 ± 0.1	12.48 ± 0.03
M4	20	21 ± 4	2.4 ± 0.1	12.51 ± 0.03

**Table 5:** Experimental results of material and mechanical properties.

Mix Symbol	Bulk Density [kg/m^3^]	Compressive Strength [MPa]	Flexural Strength [MPa]	Split Tensile Strength [MPa]	Elastic Modulus [MPa]	Poisson Ratio [-]
Base mix	2205 ± 4	53 ± 1	10.5 ± 0.3	4.12 ± 0.05	32 ± 1	0.12 ± 0.02
M1	2191 ± 4	59 ± 2	10.8 ± 0.2	4.93 ± 0.12	29 ± 1	0.12 ± 0.07
M2	2179 ± 6	62 ± 2	11.3 ± 0.2	4.98 ± 0.02	29 ± 1	0.12 ± 0.02
M3	2171 ± 5	66 ± 2	11.5 ± 0.2	5.02 ± 0.09	31 ± 1	0.12 ± 0.09
M4	2135 ± 5	68 ± 3	12.0 ± 0.3	5.06 ± 0.05	33 ± 2	0.12 ± 0.10
